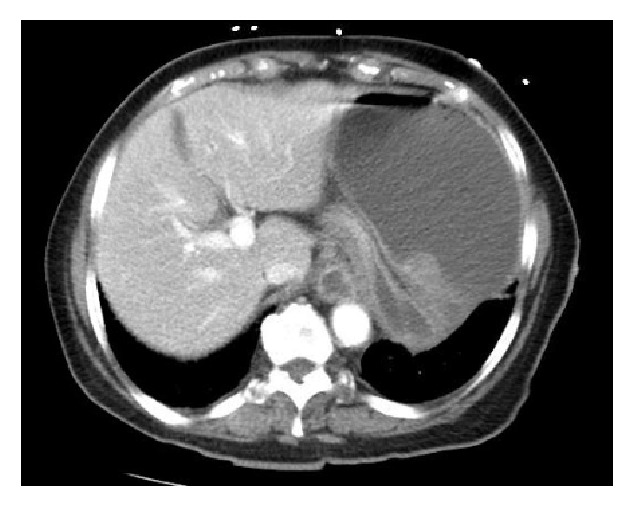# Acute Gastric Volvulus and Atrial Fibrillation with RVR: A Coincidence or Association

**DOI:** 10.1155/2017/9403601

**Published:** 2017-03-08

**Authors:** Omar Nadhem, Omar Salh, Faisal Khasawneh

**Affiliations:** ^1^Internal Medicine Department, Texas Tech University Health Sciences Center, 1400 S. Coulter, Amarillo, TX 79106, USA; ^2^Internal Medicine Department, Texas Tech University Health Sciences Center, Amarillo, TX, USA

## Abstract

Gastric volvulus is a rare and life-threatening condition that involves the abnormal rotation of the stomach around its axis by more than 180°. The association between acute gastric volvulus and atrial fibrillation with rapid ventricular response is rare with only few cases that have been reported. Our patient was an 86-year-old female who presented with upper abdominal pain, distension, nausea, and shortness of breath. Clinical and laboratory workup revealed acute gastric volvulus with diaphragmatic hernia. On presentation, she was also in atrial fibrillation with rapid ventricular response. She was successfully treated by laparotomy with reduction of the gastric volvulus and repair of the diaphragmatic hernia, with significant improvement.

## 1. Introduction

Gastric volvulus (GV) is defined as rotation of the stomach or part of the stomach on its axis by more than 180°, creating a closed-loop obstruction [1]. GV is subdivided into three types: organoaxial, mesenteroaxial, or a combination of the two. It can also be further classified as acute or chronic [2]. Classic symptoms of acute GV are known as Borchardt's triad (severe epigastric pain, nonproductive vomiting, and inability to pass a nasogastric tube) and it presents in 50% of the cases [3]. GV mimicking cardiac pain with ECG changes is rare. Reported ECG changes in the setting of GV include 1st-degree atrioventricular block, ventricular ectopy, atrial fibrillation, and inferior ST elevation [4]. The diagnosis of GV usually depends upon clinical suspicion and radiographic imaging. Abdominal plain radiographs might raise the possibility of GV. However, abdominal CT scans provide more accurate diagnosis with specific details of the anatomical abnormality [5]. As soon as the diagnosis is made, a nasogastric tube should be inserted to decrease the intragastric pressure and urgent reduction of the volvulus should be pursued to avoid acute gastric ischemia and perforation [6]. Here within, we report the case of a patient, who presented with abdominal pain and shortness of breath. She was found to have marked gastric distension and subsequently was diagnosed with acute GV and atrial fibrillation with rapid ventricular response. To our knowledge, few cases have been reported of such association.

## 2. Case Presentation

**Figure 1:**
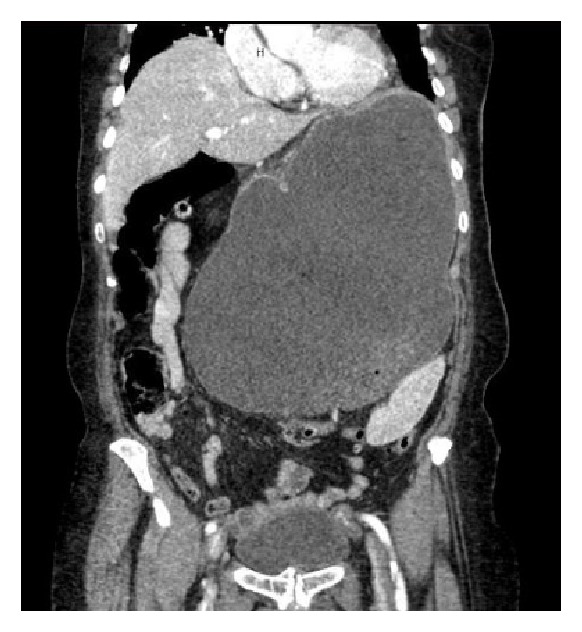


An 86-year-old white female with a past medical history significant for hypertension and hyperlipidemia presented with upper abdominal pain, distension, nausea, and mild shortness of breath for one-day duration. She denied vomiting, chest pain, fever, or abnormal bowel movement. On physical examination, her blood pressure was 206/118 mmHg, heart rate was 166 beats per minute, respiratory rate was 26 breaths per minute, and temperature was 37.3°C. The patient was alert and oriented, but in severe distress. She had clear lungs with irregular heart sounds. Her abdomen was tense, tender mainly in the epigastric region, and distended with normal bowel sounds. Laboratory tests on admission revealed hemoglobin 129 gm/L, WBC 35.5 × 10^9^/L with 89% segmented neutrophils, platelets 690 × 10^9^/L, sodium 131 mmol/L, potassium 3.2 mmol/L, magnesium 1 mmol/L, chloride 90 mmol/L, bicarbonate 24 mmol/L, BUN 7.49 mmol/L, and creatinine 88.4 *µ*mol/L. She had normal liver function test and lipase. Lactic acid was 1.3 mmol/L (high) and troponin was 0.12 *µ*g/L which then went up to 0.36 *µ*g/L. Chest X-rays showed distended stomach without abnormal lung findings. An electrocardiogram showed atrial fibrillation with rapid ventricular response and ST depression in V1–V4. Patient was started on diltiazem drip which was unsuccessful in controlling her atrial fibrillation, so amiodarone drip was added. Intravenous fluid and analgesia were given to control her pain and distress. An urgent abdominal computed tomography with intravenous contrast was performed and revealed markedly distended (Figure 1) and rotated stomach with diaphragmatic hernia consistent with gastric volvulus (Figure 2). A nasogastric tube was inserted without difficulty, and approximately 2 liters of brown-colored content was drained within two hours. Immediately after that, the patient's distress resolved with improvement of her vital signs including the atrial fibrillation which later on converted to sinus rhythm. Few hours later, patient underwent EGD that showed gastric volvulus of the entire stomach and three nonbleeding gastric body ulcers with clean bases. The duodenum was not able to be entered due to anatomic constraints. Surgery team was consulted and performed laparotomy. A large diaphragmatic hernia, gastric volvulus, and incarceration were identified. A complete gastric reduction and repair of the diaphragmatic hernia followed by gastrojejunostomy feeding tube placement were done. The patient tolerated the procedure well. Initially, she was given tube feeding, but few days later, she was tolerating oral diet. She remained in sinus rhythm and her cardiac markers trended downwards.

## 3. Discussion

GV is defined as rotation of the stomach or part of the stomach by more than 180°, creating a closed-loop obstruction [1]. The most accepted classification of GV divides it into 3 different categories: organoaxial (59%), mesenteroaxial (29%), and combined (12%), which are based on the axis around which the stomach rotates [5]. It can also be further classified as acute or chronic [2]. The exact incidence remains unknown, but the majority of the literature suggests that this condition affects either children <1 year old or adults in the fifth decade of life. Only few cases were previously reported in patients aged >70 years of age [5], with no sex preponderance [2]. The pathophysiology leading to primary GV in adults remains unclear but may involve either a laxity or a disruption of the ligaments anchoring the stomach. However, in the majority of cases, the etiology is secondary to an underlying condition such as paraesophageal hernia, diaphragmatic herniation, trauma, tumor, or phrenic nerve paralysis [6]. The signs and symptoms of acute GV include abdominal pain and distension, especially in the upper abdomen, as well as vomiting with progression to nonproductive retching [1]. GV can also cause chest pain and ischemic changes in the ECG, thereby mimicking a myocardial infarction [2]. The reported ECG changes include 1st-degree atrioventricular block, ventricular ectopy, atrial fibrillation, and inferior ST elevation [4]. The exact mechanism of atrial fibrillation in acute GV is unknown due to rarity of the reported cases. Some experimental animal studies correlate the above-mentioned association with acid-base disturbances, electrolyte abnormalities, mechanical compression of the intrathoracic stomach on the heart especially the atria, or autonomic neuropathy due to pressure of the distended stomach. On the other hand, it is known that atrial fibrillation can cause visceral ischemia due to thromboembolic mechanism. Visceral ischemia can result in volvulus, although unlikely. This theory is not much applicable in our case as the stomach has a rich blood supply. Interestingly in our patient, she converted to and stayed in sinus rhythm after decompression of the distended stomach with nasogastric tube placement, suggesting the hint of a tentative causal link.

GV is diagnosed based on the combination of suggestive history and imaging findings. Plain radiographic findings include an intrathoracic portion of the stomach, severe gastric distension, and lack of air in the distal bowel [3]; this can be followed by a barium contrast study [1]. However, in the acute setting, this may not be feasible, especially if there is diagnostic uncertainty or if the patient is critically ill. Computed tomography scan is now commonly performed, helping to delineate the anatomy and to establish any complications arising from complete volvulus [2]. Endoscopy is unreliable in the diagnosis of gastric volvulus (failure rate of approximately 68%) but can be used for the differentiation of other clinical possibilities and for gastric decompression [7]. A missed or delayed diagnosis may result in serious complications caused by the resulting obstruction, which includes strangulation, perforation, hemorrhage, ischemia, and severe necrosis [5]. Classically, a nasogastric decompression should be attempted to relieve symptoms and may result in spontaneous detorsion of the stomach [8]. The definitive treatment includes surgical restoration of normal anatomic position of the stomach with repair of any hernia defects. Several surgical procedures have been described, but the most commonly performed procedure is open reduction with or without gastropexy [8].

In conclusion, acute GV is a rare disease that requires a high index of suspicion for diagnosis and rapid surgical treatment to avoid gastric necrosis and perforation which has a mortality rate of approximately 30–50%. The presence of cardiac arrhythmias with acute gastric volvulus is rarely reported. To our knowledge, there is only one reported case of acute GV and atrial fibrillation. Physicians should be aware of potential relationship and further studies are required to determine potential mechanisms.

## Figures and Tables

**Figure 2:**